# The Significance of Justice in the Psychotherapeutic Treatment of Traumatized People After War and Crises

**DOI:** 10.3389/fpsyt.2020.00540

**Published:** 2020-06-19

**Authors:** Jan Ilhan Kizilhan, Johanna Neumann

**Affiliations:** ^1^Institute for Psychotherapy and Psychotraumtology, University of Duhok, Duhok, Iraq; ^2^Institute of Transcultural Health Science, Baden-Wuerttemberg Cooperative State University, Villingen-Schwenningen, Germany; ^3^Transcultural Psychosomatic Department, MediClin, Donaueschingen, Germany

**Keywords:** trauma, justice, post-traumatic stress disorder, war, reparation, psychotherapy

## Abstract

In the aftermath of crimes against humanity, human rights violations, and genocide, the question arises whether and how justice can be restored. A lack of social justice and continuing injustice in post-conflict areas prevent survivors from processing their traumatic experiences. As a consequence, the individuals and often their families, their community, and the whole society are changed in a lasting way. The trauma can even be passed on over generations. Yet, if war has a negative impact on health, then, programs that focus on achieving justice, peace, and stability should be able to offset or reduce this negative impact. For this reason, the importance of psychosocial well-being and mental health for the reconstruction of societies is acknowledged. Various political, legal, and social programs, like transitional justice, are being implemented in post-war regions to develop justice. Developing or restoring justice also requires good psychosocial care, like a treatment that supports individuals when coping with injustice and gaining a new sense of justice. Such a psychological treatment can make an important contribution when it comes to building new trust and improving mental health. Ethical standards in coping with trauma and developing or restoring justice in post-conflict regions are indispensable to enable long-term peace. The course for new social justice can be set, through a just health system. Thereby, only programs and legal processes, which try to do justice to the survivors and take their needs into account, are ethically justifiable. Human rights and health cannot be separated in psychotherapy with survivors of war and terror. Based on ethical principles, new approaches must be generated for psychotherapy in war regions and with survivors of war and terror. The aim will be to make an important contribution to the mental and social reconstruction of countries after mass violence.

## Introduction

In 2019, the United Nations High Commission for Refugees (UNHCR) registered 70.8 million people who had been forcefully displaced. Out of these, 25.9 million were classified as refugees who had been forced to leave their homeland on account of persecution, war or violence (1). The consequences of prolonged exposure to conflict and persecution are frequently exacerbated by displacement and deprivations. This, in turn, increase the refugees' vulnerability to many mental health problems (2). As consequences of the traumatic experiences, post-traumatic stress disorder (PTSD) and depression are the most common mental health problems among refugees. One of the largest meta-analysis with refugees and other survivors of torture and war from over 40 countries suggests a prevalence of 30.6% for PTSD and 30.8% for depressive disorders (3). It is supposed that the number is a lot higher in displaced people, who live in countries with ongoing violence and a bad supply situation. Prolonged exposure to conflict and persecution and protracted conditions of deprecations and displacement are likely to increase the prevalence.

Medical doctors and psychologists who work with these people agree that some form of justice must be achieved to process what has been experienced. “No healing without justice” says Dr. Mukwege, Nobel Peace Prize laureate 2018, about his work with women and children who survived sexual violence in Eastern Congo. Referring to the victims of ISIS in Northern Iraq, psychotraumatologist Prof. Kizilhan and Nadia Murad, the other 2018 Peace Prize laureate, emphasize that the psychological wounds of women can only be healed if they are also given legal justice (4).

Yet, in many conflict areas, reparations, rehabilitation measures, and the prosecution of war crimes are only implemented after many years or not at all. Often national governments are not interested in pushing forward the right for justice of minorities, especially if they were involved in the conflict themselves. The international community and the International Criminal Court (ICC) are needed to intervene. Yet, their possibilities are often limited, especially when the affected country has not signed the Rome Statue. The Rome Statute is a treaty (1998) that allows the ICC to prosecute war crimes, crimes against humanity and genocide in the signed countries and to bring them to justice. But the harmed communities cannot wait years to have their desire for justice addressed (5).

There are further challenges. Neither legal compensation nor the prosecution of perpetrators is enough to cause effective justice. Those do not automatically help individual survivors or the collective community heal (6). Especially, when their demands and their cultural and societal background are not taken into account. Thus, a new, transcultural justice approach is needed to help individual survivors and harmed societies heal after mass atrocities.

Understanding how justice can be established or restored in conflict areas and war-traumatized societies, means, applying basic ethical standards. Equal access to health care, as a form of justice, is one of the main principles of biomedical ethics. Consequently, restoring justice has to include the accessibility of health services. Mental health and support have to be addressed as much as physical health in this context, to increase the changes for long-term improvements.

Most psychological concepts of justice were developed and tested in Western countries and were discussed in terms of social inequalities. Thus, there is an information gap on the consequences of perceived injustice among survivors of war, mass violence, and genocide in non-Western societies (7). The understanding of justice and the ability to cope with injustice cannot be generalized. Culture, religion, the experiences of one's ancestors, and belonging to a persecuted minorities shape the perception of justice and the ability to cope with injustice.

The following article addresses the absence of justice and discusses its impact on individuals and societies that were affected by war and mass violence. Focusing on Middle Eastern minorities and the aftermath of the ISIS terror in 2014, the article examines justice programs and their effect on harmed individuals in post-conflict areas. Demands for psychotherapy programs for survivors of gross human rights violations are elaborated, to include coping with the experienced injustice (8).

## The Aftermath of Violence

Violence leads to long-term physical, social, and psychological consequences for survivors and their families. This happens especially when socio-economic, political, religious, or ethnic discrimination continues after the conflict, and adequate health care is not provided (9–12). Persistent bad conditions, like lacking hygiene facilities in overcrowded camps, are keeping the risk of threats for people's health high, even after the end of conflicts. Physical consequences like dismemberments and the loss of walking or internal injuries, especially after sexual violence, as well as widespread malnutrition and weakened immune systems will only heal, if there is an immediate access to healthcare. Otherwise, survivors will likely experience everlasting problems.

### Psychological Impact

Experience shows that about 50% of severely war-traumatized people develop trauma sequelae, of which about 25% become chronic (13). The most common psychological problems resulting from mass atrocities and war events are depressive disorders and PTSD (1, 14). Yet, there is a lack of data on the prevalence of mental health disorders among populations living in protracted displacement situations, especially in conflict-affected middle-eastern countries (1). In a study of Syrian Kurdish refugees in the Kurdistan Region of Iraq, almost all participants had experienced at least one traumatic event, while 86.3% had experienced three or more traumatic events. The prevalence of PTSD and the prevalence of depression were both about 60% in that population (15).

Prevalence is estimated higher among survivors of rape, military action, captivity, internment for ethnic or political reasons or genocide (16). In a random sample of female survivors of the Rwandan genocide, researchers found a prevalence rate for PTSD of 58% (17). In a random sample of women affected by sexual violence in former Yugoslavia, the prevalence rate for depression amounted to 80% (18). The prevalence rate for PTSD (58%) and depression (55%) among female Yazidi women was found to be very high even 5 years after they had survived the 2014 ISIS genocide and captivity (19). This supports the assumption that about 71% of refugees who fled from their homeland and who have depression also suffer from PTSD (20).

Apart from mental disorders, there are many other psychological consequences reported by survivors. Dead and missing family members lead to grief and worries, especially in collective societies. Often, the social support system is destroyed and connections to neighbors and other groups in the societies are harmed by feelings of mistrust and hate (21). In addition to the lack of health service, the lack of education, employment, and shelter cause people to feel loss of control over life and security (22). Among people in refugee camps, daily stressors like the continuing concern for safety and a lack of basic resources, like water, shelter, and food, can exacerbate mental problems (23). A study among stateless Rohingya refugees in Bangladesh showed that the daily environmental stressors of living in the camp partially mediated the direct mental health effects of trauma exposure that were found (24). Furthermore, these upholding instabilities reduce the chance of a long-term recovery for individuals, their communities, and the entire society. For that reason, refugee camps should only be a short-time resolution.

## Justice and Injustice in the Aftermath of Violence

One popular cognitive concept of justice is the idea of a belief in a just world (25). According to this theory, people generally believe that the world is a just place in which just things will happen to them. They assume that there is a reason why people experience injustice. Yet, when one experiences extreme violence like rape or other war crimes, or natural disasters, serious accidents, or the sudden loss of loved ones, this idea of a just world can get shattered (26). People, who hold on to the image of a just world want to understand why they experienced injustice (25). If they come to the conclusion that they must have done something wrong to deserve the injustice, they may react with feelings of guilt, desperation, or self-blame (27). Others, who cannot grasp that their assumptions of a just world do not stand, may react with embitterment (28).

So far, most of these assumptions were developed through studies with survivors of accidents and other non-man-made disasters (26, 29, 30). It is known that trauma that is intentionally evoked have a much higher damage potential. They are more likely to lead to severe stress reactions than accidental ones. The particularity of man-made disasters seems to be the extreme power gap and that the destruction is usually done with full intention in order to humiliate the other person or, in the case of torture, to destroy their personality (31).

Furthermore, most of these ideas are based on Western concepts of justice, rightfulness and self-worthiness. For this reason, they cannot directly be transferred to the perspective of survivors from other cultures and religions, e.g., African or Middle Eastern. In collective societies, not only the personal experiences of injustice matter but also unjust experiences that happened to other members of the society or ancestors play a central role in one's perception of justice (32). With regard to shame and guilt as reactions to traumatic experiences, societies vary a lot (33). The ideas of reconciliation and revenge are influenced by culture and tradition. Different, traditional acts of reconciliation exist, and these have to be considered.

Despite these individual and cultural differences, atrocities and human rights violations can be generally seen as actions of injustice (5). In the aftermath of violence and war, these actions of injustice have to be addressed to give survivors back a sense of justice. It can be assumed that the long-term consequences of violence are often perpetuated by continuing injustice after the official end of conflicts. Low access to resources, persistent unjust treatment, and a lack of possibilities to restore justice maintain the trauma of many survivors.

Psychiatric disorders and mental health problems become chronic over time. This, in turn, hampers medical or psychological treatments. Children, families and social relationships in these communities become affected in consequence (22, 34, 35). When no interventions are set in place, the trauma and the feeling of injustice can be passed on over generations. In this way, they can weaken and change whole societies forever (36). Especially, when not only individuals but also whole communities are affected. This is most visible in survivors of genocide, such as Holocaust survivors, or Yazidi genocide survivors.

More research is needed to understand the reaction and needs of survivors from specific areas and of varying disasters. Then, once developed concepts for the restoration of justice, like transitional justice programs, have to be tailored to the respective cultural and regional characteristics.

### Transitional Justice

Ordinary national legal systems in which public persecutors pursue individual offenders are not a sufficient response to mass atrocities and unable to restore justice for all the victims. Transitional justice refers to the ways in which countries emerge from conflicts, repression or systematic human rights violations, which are so numerous and severe that normal justice systems are not able to provide an adequate response (37). It is seen as an opportunity for reconciliation and prevention of future human right violations.

In this context, reconciliation is understood as a large-scale process with many aspects and approaches (38). The aim is not necessarily forgiveness (39), but the possibility for different individuals, parties, or peoples to live together or next to each other in peace, confronting their past (38). Nadler and Shnabel (40) define it as ***“***the process of removing conflict-related emotional barriers that block the way to ending intergroup conflict***”***.

The aim of all transitional justice processes is respecting and installing individual and collective rights and, most importantly (41), the prevention of future human rights violations. So far transitional justice mechanisms have been implemented in more than 90 countries with varying degrees of success. The gacaca, the Rwandan village tribunals for truth and reconciliation after the 1994 genocide, are often cited as a successful example of transitional justice (6).

There are four main approaches for transitional justice: The criminal prosecution of at least some of the most responsible for the most severe crimes; truth-seeking processes by non-judicial bodies, like the truth committees in South Africa; individual, collective, material, or symbolic reparations for human rights violations; reforms of laws and institutions, including police, judiciary, and military and approaches to restore new confidence in those (42). These different approaches should not be seen as alternatives for one another, but be combined according to the needs of the respective society.

For example, monetary compensation, although necessary, cannot be viewed as the only means for repairing the wrong done to the survivors. For this reason, prospective reparation programs should include moral or symbolic reparations, in addition to pecuniary redress and monetary benefits (43). Symbolic reparations, for example, aim at addressing less tangible harms arising from serious violations of international law. They may take several different forms such as apologies, memorialization, or truth-seeking (41, 44). Great potential is seen in the fact that they carry meaning and thus can help victims in particular and societies in general to make sense of the painful events of the past (44, 45).

Transitional justice should help with putting the victims and their dignity first, making sure that ordinary citizens are safe in their own countries, protected from abuses of their authorities and violations by others (37). For this reason, reparation and justice programs must be designed with the participation of survivors and relevant civil society actors and groups. Participation is also important to ensure that reparations are accessible, equitable, effective, and strengthen the agency and empower victims, as well as to strengthen awareness of rights (37).

However, an exact evaluation of these approaches through a meta-analysis is still missing. There are hardly any studies that examine the collective and individual satisfaction with the outcomes of transitional justice programs. Some studies show that, frequently, individual survivors do not agree with the reported success of these programs. For example, one survey reported that survivors in Croatia and Bosnia and Herzegovina were not supportive of the ICTY, although it was said the trials were for the victims and to provide reconciliation (46). Corkalo et al. (47) describe how all different national groups there felt that their group was the greatest victim and the ICTY was against them. Another study even suggested that the ICTY increased the hostility between the ethnic groups in Bosnia and Herzegovina instead of improving societal peace (48). On a political level, critics argue that pursuing justice in the midst of ongoing conflicts can have a domino effect. They argue that it can interfere with the delivery of humanitarian aid, ongoing peace negotiations, and agreements, especially when powerful actors can block such an agreement, because they may fear punishment for past actions or have not signed the Rome status, like Syria or Iraq (49).

Transitional justice could promote social reconstruction and mitigate the consequences of trauma, as long as the right steps are taken and its outcome is closely monitored (50–54). Yet, further research and analyses are needed to understand the outcome of such programs for the affected individuals and societies, with regard to their physical and mental health.

Additionally, studies have shown that economic improvements and improvements in the rule of law have a positive impact on several health indicators, like life expectancy and reduced child mortality. In other words: Improvements in the rule of law lead to better health outcomes, either directly or indirectly through improvements in the level of development (55). Thus, economic support and prospects for the future often lead to better coping with the trauma and can, therefore, support the transitional justice process and increase the willingness for long-term reconciliation and peace (55).

## Justice and Mental Health

### The Mental Health Outcome of Justice Initiatives

The implementation of transitional justice mechanisms have sparked a lively debate among some psychiatrists, lawyers, anthropologists, and international and local NGOs on how traumatic experiences shape the ability of individuals and groups to respond to transitional justice initiatives (35, 56). Yet, there are only a few empirical studies that have investigated the link between transitional justice and mental health (22, 57–59).

So far, existing studies have investigated how law initiatives affect the health of individuals and communities (34, 55, 60), social healing (61), and deterrence of violence (62). Yet, these studies have not revealed an effective relationship between the processes and mental health. This might be due to the methodology of the studies. There is a lack of standardization in how the various independent and outcome factors are defined and investigated (i.e., exposure to the trauma events, assessment of symptoms of PTSD, individual vulnerability to mental illness, resilience, reconciliation, revenge, forgiveness, etc.).

Yet, some of these studies challenge the claim that truth-telling has a healing effect for individuals, although it plays a pivotal role in post-conflict reconciliation processes around the world (6, 57, 59). For this reason, we assume that a difference between individual psychological healing and societal healing must be made when elaborating the outcome of such efforts. For example, on a societal level the *gacaca* in Rwanda seems to have helped the society to move forward (6). Yet, less is known about the psychological and mental health effects of this program on the individual survivors.

On an individual level studies demonstrate that traumatic exposure and PTSD symptoms are associated with attitudes toward justice and reconciliation (56). Some studies prove a reduction of PTSD symptoms in traumatized people through reconciliation programs in general (36), while others show that a direct contribution of one's sense of justice toward PTSD symptoms exists (64).

On the other hand, it is shown that people with less openness to reconciliation, and more feelings of revenge, show higher PTSD symptoms (34). In many societies, mental health problems and feelings of anger or revenge are very common in survivors even years after the traumatic events (65). This impedes social coexistence and successful peace-building processes and calls for actions on several levels to install a sense of justice (65).

### Justice in Psychotherapy

The question arises on how psychotherapy, if available, can contribute to the restoration of justice. In general, psychotherapy in post-conflict zones, refugee camps, and psychotherapy with people from different cultures should follow a culturally sensitive approach (2, 7). Especially in therapy sessions with severely traumatized people, it is important to consider their cultural and religious resources, coping strategies, and often their family system. Apart from the client's personal consequences and experiences, the psychotherapist must consider the following questions when working with clients from collective cultures. What does the event mean to the client's family and social system? How does it influence the way they behave to the client? (19).

Most of the concepts for therapeutic trauma interventions suggest that people need stabilization, orientation and security to be able to start a therapy. To assure that people can get involved in a therapeutic process, public health services must be quickly reconstructed and incorporated into justice programs. The main public health and medical goals in post-conflict areas have to be the treatment, reduction, and prevention of disease, mental illness, disability, and premature death.

A common goal of promoting and protecting individual and societal physical, mental, and social well-being can give survivors the impression that the country and the government are making a serious and long-term effort to care for them (66) and are trying to make amends for the injustices. Such recognition is closely related to the WHO's view of health, which defines health as a state of complete physical, mental, and social well-being and not merely as the absence of disease (67). For this reason, we suggest that health care and the restoration of justice (political, social, psychosocial, and medical) are closely linked to improving the health situation of survivors.

The existence of justice initiatives, including health programs, enables psychotherapy to focus on the actual trauma therapy process. Refugees cannot wait years to have their desire for justice addressed by the international community or local institutions (5). However, therapy in conflict zones must address the lack of justice initiatives and the feeling of injustice, as the ideal scenario is rarely the case.

So far, there are some cognitive behavioral trauma manuals which are specifically designed for survivors of severe human rights violations in general (68). This includes psychotherapeutic or psychoeducative programs and interventions that were tailored for specific post-conflict communities. Furthermore, some interventions for groups who are targets of systematic injustice, like Black communities in the US, have been developed to empower those affected (69). These existing ideas should be customized, integrated and expanded for a new psychotherapeutic justice approach. Tailored to survivors of gross injustice in post-conflict and crisis areas, this approach should provide a supplement to general mental health support and trauma therapeutic interventions.

Such a new approach could include empowerment strategies as well as rebuilding trust in people and social bonds. New waves of violence can be a long-term consequence of an upholding sense of injustice (56, 70). Thus, it is necessary to address reconciliation, in addition to legal procedures. According to Pham et al. (56), people might only be able to accept and promote social justice if they build trust. A shared vision and future can emerge by creating social bonds with sufficient economic and health care (56).

According to Range et al. (69), psychotherapy with members of traumatized communities should empower those who have been disempowered, by acknowledging their strengths and cultural resources. Psychotherapists can apply cultural empowerment by asking “what would right this awful wrong?” (69), supporting their clients to be self-advocating. Resources and successful coping mechanisms might be found, among others, in people's cultural and traditional heritage, and family systems.

As previously discussed, most people in post-conflict areas and refugee camps keep facing acts of injustice and instability after the main traumatizing event. If the required systematic changes of their situation do not happen, therapy should help with the acceptance and dealing with everyday life as well. Therapy can counteract these feelings of injustice, exhaustion, bitterness (28), or anger by making use behavioral strategies. This is needed, because studies found anger to be a negative predictor for PTSD outcomes in traumatized survivors (71). Yet, with some patience, acceptance of the unjust situation has to be developed.

Furthermore, the therapy itself can help people to achieve a sense of justice. For example by recognizing their suffering, the therapist can try to fulfill some of the survivors' demands for justice in the therapeutic setting. Going further, some authors emphasize that a psychotherapist's work is not limited to the therapeutic setting when they work with people who have survived actions of injustice. In this point of view, psychotherapists should get personally involved in actions for justice (69).

Some researchers argue that forgiveness should also be considered in therapy, as it allows survivors to regain control, leave their victim status, and experience themselves as self-effective (72). Positive connections between forgiveness and mental and physical health have been replicated several times (72). More forgiving people seem less likely to suffer from depression, anxiety, and anger, have lower cardiovascular vulnerability and better self-esteem than those who are not forgiving (72). Yet, forgiveness is only likely to appear, if a sense of justice exists and, therefore, a successful justice initiative is a prerequisite for it.

In this context, we suggest that specific cognitive behavioral modules have to be developed and tested. These modules have to be based on the needs of the affected groups, addressing the sense of injustice in post-conflict therapy to improve mental health.

In the following paragraph, we introduce a theoretical framework for health and justice implementations after gross human rights violations and war. This framework will allow, among others, psychotherapeutic mental health interventions.

### Framework and Model

**Figure 1:**
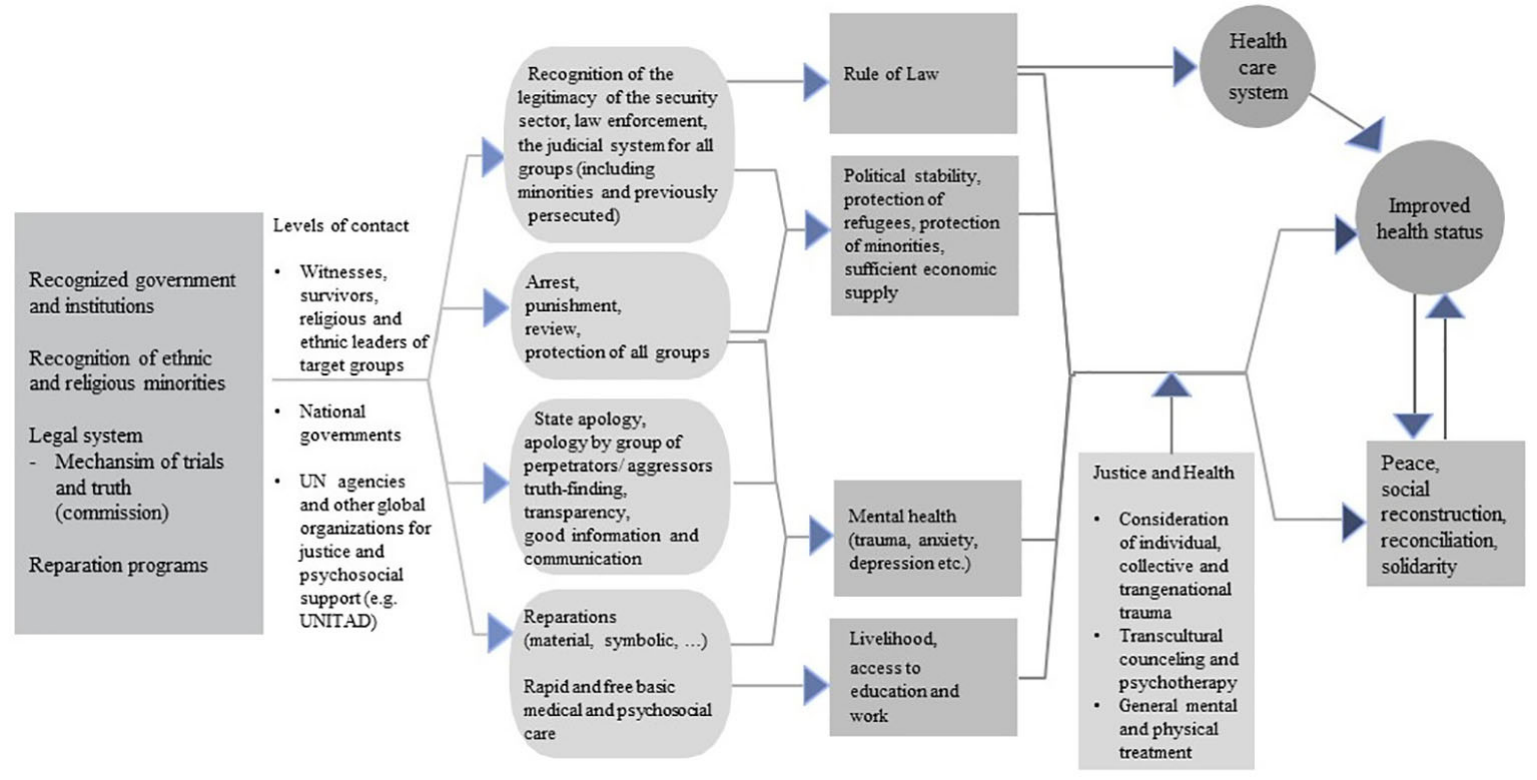
Steps toward improving health and justice after mass violence and collective human right violations in post-conflict and crisis areas.

To restore or develop justice in a society after mass violence and gross human rights violations committed by various actors, internal (state, military, ethnic, and religious groups, terrorist organizations) and external (foreign states, external terrorist organizations, etc.) steps must be taken on many levels. The individual survivors, witnesses, leaders of the affected group, as well as national and international agencies and the relevant governments have to be included in that process to assure that the result fits the cultural concept of justice and meets the expectations of the survivors and their organizations. The active participation of the affected people is urgently needed to increase the chances to successfully achieve change in the society and the physical and mental status of the individuals. **Figure 1** shows how different kinds of justice initiatives and tools must be set in place in order to enhance individual and collective health in affected regions, and the chances for peace, social reconstruction, reconciliation, and solidarity.

Recognition and consideration of all the involved and affected parties must be followed by investigations of crimes, which should be shared at national and international forums. Restoration of justice in the process of transitional justice must be defined and criteria must be established in accordance to the survivors' expectations. In a further step, the effects on health (psychosocial and medical) and social structures of all those involved must be investigated.

Subsequently, a political and legal basis must be created so that health practitioners can implement psychosocial, psychotherapeutic, and medical intervention programs including the support and implementation of national and international humanitarian aid programs. At the same time, political decision-makers have to develop long-term mechanisms to prevent violence, for example, between the various conflict parties in post-conflict situations or between the various ethnic and religious groups in a country.

Empirical data from nationwide health programs should be made available for programs to achieve or restore a sense of justice, to identify patterns, and to reveal the systematic nature of violence. In order to demonstrate the necessity of these interventions, one can collect data on the impact of human rights violations and mass violence on health, drawing on retrospective cross-sectional studies and informational monitoring systems. Health outcome measures include mortality, injuries from violence, disability, morbidity, and mental illness (73). This allows patterns to be identified and can reveal the systematic nature of violence.

Furthermore, economic improvement and improvement of the rule of law in the affected region should be supported as soon as possible, as they support positive health outcomes and better coping with the trauma. Additionally, they can increase the chances for long-term peace (55).

## Case Study Iraq—Isis Survivors

The genocide of ISIS against Yazidi in Iraq and Syria and the mass violence of this terrorist organization against other population groups led to a psychosocial breakdown in the entire society and thus to a dramatic decline in mental health. The fact that this genocide was perpetrated after decades of dictatorship and mass violence by the Saddam regime in Iraq and the Assad regime in Syria accelerated this decline. While, for example, the prevalence of various disorders in Iraq in 2006 was still comparable to that of the world population, current figures are different (74, 75). In the high-risk cities of Ramadi and Fallujah, which were occupied by ISIS, 55.8% of the population between 12 and 23 years of age suffered from an affective disorder and 63.4% from PTSD (76). In comparison, the prevalence of affective disorders in the Iraqi population in 2007 was 3.99% and the prevalence of PTSD amounted to 1.63% (76).

The systematic sexualized violence against the Yazidis has traumatized both the victims and the traditional patriarchal society in which the violation of female sexuality is both a collective violation of honor and humiliation. This change will presumably keep the medical-therapeutic field busy for generations to come. However, various forms of sexualized violence did existed before the invasion of the ISIS in Iraq (77). It is well known that in collective and traditional societies such as Northern Iraq, issues such as sexualized violence and torture are often concealed and tabooed to prevent dishonor or exclusion from the community (78). Disclosure of the abuse can lead to dishonoring the patient, but also the whole family. Consequences could be difficulties in finding a spouse (for the patient and her female siblings) or the murder of the perpetrator (honor killing) or, in case of indirect blame, the murder of the patient. For this reason, the consequences of revealing abuse may be more serious for the patient than continuing to bear it (79).

It is important to understand the connection between the exposure of violence and traumatization in the specific social context. Affected people need to get a psychotherapeutic treatment that takes into account their cultural background and their personal and collective sense of injustice. Thus, the consequences might affect them throughout their life if these and other forms of violence remain untreated due to a lack of psychotherapeutic care.

Psychosocial support is one aspect of rebuilding the society and responding to the desire for justice. Yet, to rebuild these societies in a just way, a combination of many justice initiatives is needed. Cross-sectional surveys can be designed to assess attitudes of the affected population toward justice mechanisms such as symbolic reparations, restitutions, truth commissions or amnesty provision (56). With regard to attempts of rebuilding these societies two aspects are essential. First, one must understand how traumatic experiences affect the ability of individuals and groups to respond to justice, when available. Secondly, one must understand how these initiatives, in turn, affect the health of individuals and communities.

A coalition of over 20 Yazidi NGOs has collected all the demands that female Yazidi survivors from Iraq have for just reparations (80). If Iraq could fulfill these demands with international help, a feeling of justice in survivors might be achieved. This, in turn, would also improve their personal and collective mental health. Fulfilling these demands is synonymous with the establishment of many actions emphasized in our introduced model. Several services and aspects are important in this context. It is crucial to establish rehabilitation and compensation services. Moreover, one must guarantee a non-repetition, drawing on the rule of law. These measures, in turn, would have numerous positive results. First, mental and physical health of those affected might improve. Secondly, members of the community might be able to move back in their original areas one day. This includes that they feel safe enough to live in peace eventually. This requires individual and collective replications as described in **Box 1**.

Box 1 **Individual and collective reparations.**

**Reparations**

Provide a mix of individual and collective reparations in the prospective reparation program(s). This is because both victims and specific communities were targeted.

Monetary compensation, which is necessary, cannot be viewed as the only means for repairing the wrong done to the victims. For this reason, prospective reparation program(s) should include moral or symbolic reparations, in addition to pecuniary redress and monetary quantifiable benefits

**Restitutions**

Undertake measures with the principal aim of restoring the victim, as far as possible, to a position occupied before the violations of international human rights or humanitarian law occurred. Measures should include: protect security to return, restoration/rebuilding of private property and of public infrastructure, reinstatement to employment, […]

**Compensations**

Compensate victims for any economically assessable harm caused by violations of human rights and humanitarian law including physical and mental harm, lost opportunities, material damages, loss of earnings (including the loss of earning potential and moral damages) to the maximum of available resources (43). The amount of compensation should reflect the gravity of violations.[…]

**Rehabilitation**

Provide a range of rehabilitation services to victims (link) and, if required, their family members, meeting relevant quality standards and within physical proximity to those areas where victims reside. These services should go beyond medical and psychological care to encompass at least: social services, legal services, education opportunities, support for mothers with children of rape, vocational training

**Satisfaction**

Offer a range of non-monetary or symbolic measures specifically designed to afford satisfaction to the victims such as: ensuring adequate participation of victims in any criminal proceedings relating to violations committed against them; identifying remains of all deceased persons; recognizing that certain events amounted to international crimes (war crimes, crimes against humanity, genocide); memorialization efforts; satisfaction measures should be implemented in accordance with the traditions and sacred rituals of affected communities

**Guarantees of non-repetition**

Undertake a range of measures aimed at making sure that similar crimes will not happen again such as: ensuring that all the perpetrators of gross human rights violations (link) are prosecuted and adequately sentenced; stop extrajudicial punishment and discrimination of individuals and families with alleged ISIS ties; acceding to the Rome Statute of the International Criminal Court (ICC); […]

## Discussion

We assume that health inequalities are rooted in social injustices (81). Furthermore, we suggest that peace, stability, and justice might offset or, at least, mitigate negative health consequences of war and injustice. This requires programs that use the means of the law to do justice to survivors and provide social, psychological, and medical care nationally and internationally. In this way, they can be quickly and effectively reintegrated into society and receive all the rights and duties of an equal citizen. This is only possible through health.

The ethical significance of the universal human rights should be indisputable; for this reason, efforts to restore justice after gross human rights violations, like torture and genocide should be obligatory. In article 25 of the Universal Human Rights Declaration it is stated that “[e]veryone has the right to a standard of living adequate for the health and well-being of himself and of his family, including food, clothing, housing and medical care and necessary social services, [… ]” (82). Moreover, justice, meaning among others, equal access to medical support and health services is one of the major ethical principles in bio-medical ethics (83).

Thus, when approaches of justice are made, health care services including mental health care services should be enrolled. The long-term effects of untreated PTSD and other mental disorders are severe. Experienced, untreated trauma often prevents reconciliation and peace processes and can even be passed on to the next generation (36, 70), resulting in further destabilization and conflict in the affected societies.

Health outcomes usually provide a good measure of political processes and of the programs that aim to respond to the conflicts (9, 84, 85). According to Salama et al. (12), traditional health interventions, for example vaccination campaigns, clinical treatment, water, and sanitation, alone have limited health benefits and cannot sufficiently minimize the impact of war on society. But still, ethically responsible action includes promoting traditional measures that support the health of many people, such as ensuring access to clean water or clean air (86), as well as health services for the affected individuals.

If war-traumatized people experiencing injustice are to learn how to deal with their traumatization and to participate in society, new ethical standards have to be considered when implicating justice, reconciliation, and reconstruction programs. Psychotherapy and psychosocial support play an important role in these processes. They can be secure places in times that are shaped by insecurity and ongoing injustice. Symbolic collective reparations and other transitional justice mechanisms have to include the survivors' voices and demands. In therapy, they can learn to formulate those and to find their voice to call for justice. People can also develop strategies to accept and move on from unjust circumstances that cannot be changed. Furthermore, psychotherapy, especially trauma therapy, can help decrease the prevalence of mental health problems. This, in turn, can increase the stability in individuals and the society.

In conclusion, in the field of psychotherapy, the development of modules on justice and mental health and their evaluation are necessary for both individual and group therapies,

All of the previous considerations support the assumption that justice after massive human right violations and atrocities should not only be discussed from a judicial view. Instead, an interdisciplinary approach including law, social service, medical, psychological, and political professionals is needed when developing justice initiatives, like transitional justice programs. When doctors, therapists and counselors work with survivors of war and terror, a minimum of knowledge about the principles of humanitarian law and justice is necessary. The same applies for bureaucrats and political decision-makers in the health care system.

Furthermore, scientific research, especially in the context of war and mass violence, must discuss ethical questions that address entire societies, in addition to individual patients.

In our globalized world, ethical principles cannot be limited to national borders. They are meant to be applied internationally (86). Hence, national and international players must be included in the development of such programs, particularly, when the government is involved in the conflict. When international researchers and professionals are involved, it must be ensured that the developed programs fit the targeted cultures and societies, instead of imposing, e.g., Western concepts on collective Middle Eastern societies (87). Therefore, a culture sensitive approach is needed.

## Author Contributions

JK and JN wrote the article. All authors contributed to the article and approved the submitted version.

## Conflict of Interest

The authors declare that the research was conducted in the absence of any commercial or financial relationships that could be construed as a potential conflict of interest.
